# Carbonic Anhydrase 1-Mediated Calcification Is Associated With Atherosclerosis, and Methazolamide Alleviates Its Pathogenesis

**DOI:** 10.3389/fphar.2019.00766

**Published:** 2019-07-10

**Authors:** Lin Yuan, Minghua Wang, Tianqi Liu, Yinsheng Lei, Qiang Miao, Quan Li, Hongxing Wang, Guoqing Zhang, Yinglong Hou, Xiaotian Chang

**Affiliations:** ^1^Medical Research Center of Qianfoshan Hospital Affiliated with Shandong University, Jinan, China; ^2^Cardiac Surgery Department of Qianfoshan Hospital Affiliated with Shandong University, Jinan, China; ^3^Medical Research Center of the Hospital Affiliated with Qingdao University, Qingdao, China; ^4^Cardiology Department of Qianfoshan Hospital Affiliated with Shandong University, Jinan, China

**Keywords:** carbonic anhydrase 1, calcification, atherosclerosis, acetazolamide, methazolamide

## Abstract

Vascular calcification is an important pathogenic process in atherosclerosis (AS); however, its immediate cause is unknown. Our previous study demonstrated that carbonic anhydrase 1 (CA1) stimulates ossification and calcification in ankylosing spondylitis and breast cancer. The current study investigated whether CA1 plays an important role in AS calcification and whether the CA inhibitor methazolamide (MTZ) has a therapeutic effect on AS. We successfully established an AS model by administration of a high-fat diet to apolipoprotein E (ApoE^−/−^) mice. The treated animals had significantly increased serum levels of high-density lipoprotein cholesterol (HDL-c) and nitric oxide (NO) and decreased serum concentrations of total cholesterol (TC), triglycerides (TG), low-density lipoprotein cholesterol (LDL-c), interleukin (IL-6), interferon (IFN)-γ, granulocyte-macrophage colony-stimulating factor (GM-CSF), tumor necrosis factor-α (TNF-α), chemokine (C-X-C motif) ligand 1/keratinocyte-derived chemokine (CXCL1/KC), and C-C motif chemokine ligand 2 (CCL2)/monocyte chemoattractant protein 1 (MCP-1). The treated mice also had reduced AS plaque areas and fat accumulation, with no clear calcium deposition in the intima of the blood vessels. CA1 expression was significantly increased in the aortic lesions, particularly in calcified regions, but the expression was dramatically lower in the mice that received MTZ treatment or MTZ preventive treatment. CA1 was also highly expressed in human AS tissues and in rat vascular smooth muscle cells (VSMCs) with β-glycerophosphate (㒐β-GP)-induced calcification. Acetazolamide (AZ), a CA inhibitor with a chemical structure similar to MTZ, markedly suppressed calcification and reduced CA1, IL-6, IFN-γ, GM-CSF, and TNF-α expression in cultured VSMCs. Anti-CA1 small interfering ribonucleic acid (siRNA) significantly suppressed calcification, cell proliferation, and migration, promoted apoptosis, and reduced IL-6, IFN-γ, GM-CSF, and TNF-α secretion in cultured VSMCs. These results demonstrated that CA1 expression and CA1-mediated calcification are significantly associated with AS progression. MTZ significantly alleviated AS and suppressed CA1 expression and proinflammatory cytokine secretion, indicating the potential use of this drug for AS treatment.

## Introduction

Vascular calcification is involved in the plaque formation of atherosclerosis (AS) (Ross, 1999; Gamble, 2006; Cianciolo et al., 2010). Arterial calcification is an active, cell-regulated process occurring during osteogenesis and includes the transition of vascular smooth muscle cells (VSMCs) to osteoblasts and to the crystallization and precipitation of hydroxyapatite salt (Adeva-Andany et al., 2015). Runt-related transcription factor 2 (Runx2), bone morphogenetic protein 2 (BMP2), and alkaline phosphatase (ALP) interact and influence each other, leading to a process similar to that of osteoblast-like differentiation (Aikawa et al., 2007). However, the immediate cause of calcium salt deposition is unknown.

Carbonic anhydrase 1 (CA1) is a member of the carbonic anhydrase (CA) family that reversibly catalyzes the hydration of CO_2_ to form HCO_3_^−^, which then rapidly binds to calcium ions to form calcium carbonate (Supuran, 2008). We have found that CA1 expression is specifically upregulated in the synovial membrane in patients with ankylosing spondylitis (Chang et al., 2010). The most distinctive pathological manifestations of ankylosing spondylitis are inflammation of the hip and spinal joints, hyperosteogeny, joint fusion, and fibrosis (Zhang et al., 2003; Landewe and van der Heijde, 2009). We then found that CA1 could promote joint calcification, ossification, and joint fusion by accelerating calcium carbonate deposition (Chang et al., 2012; Zheng et al., 2012). Additionally, our group found that CA1 was highly expressed in breast carcinoma tissues and in blood from patients with breast cancer, leading to the calcification of the tumor tissue, inhibition of apoptosis, and promotion of tumor cell migration (Zheng et al., 2015). Thus, CA1 plays important roles in promoting biocalcification. Acetazolamide (AZ) and methazolamide (MTZ) are CA inhibitors (Masini et al., 2013; Solesio et al., 2018). MTZ and AZ are sulfonamide derivatives and are clinical drugs used in the treatment of glaucoma. MTZ is an improved version of AZ with a relatively low adverse reaction rate (Mincione et al., 2008). As shown in our previous studies, the induction of calcification in human osteosarcoma Saos-2 cells and murine mammary adenocarcinoma 4T1 cells upregulated CA1 expression. Treatment of these cells with AZ not only reduced CA1 expression but also suppressed cell calcification (Chang et al., 2012; Zheng et al., 2015). Our clinical study showed that MTZ was an effective treatment for patients with active ankylosing spondylitis by suppressing CA1 expression and joint fusion (Chang et al., 2011).

CA may play important roles in AS. As shown in the study by Oksala et al., CA2 and CA12, members of the CA family, were highly expressed in human atherosclerotic plaques and might be associated with osteoclast-like cells of a mononuclear cell lineage in patients with advanced AS and were involved in plaque remodeling (Oksala et al., 2010). Ando et al. examined abdominal aortic aneurysm using proteomics and detected an abundant CA1 autoantigen, suggesting an important role for CA1 in the formation of this lesion (Ando et al., 2013). Based on their findings and the results reported by our group, we hypothesize that CA1 plays an important role in the AS process by stimulating tissue calcification and that the CA inhibitors AZ and MTZ can treat AS by inhibiting CA1 expression.

This study investigated the function of CA1 in AS and its underlying mechanism. We first examined the expression of CA1 in human AS tissues and in the aortic tissue of an AS mouse model to determine the relationship between CA1 expression levels and AS pathogenesis. Next, we treated the AS animal model with MTZ to determine the therapeutic effect of the CA inhibitor on AS and AS plaque formation *in vivo*. Furthermore, we cultured rat VSMCs to observe CA1 expression after calcification induction. We then treated VSMCs with anti-CA1 small interfering ribonucleic acid (siRNA) or AZ to confirm the role of CA1 in VSMC calcification *in vitro*. MTZ is a clinical drug, but no sterile MTZ for cell culture is available. Sterile AZ for cell culture experiments is commercially available, but clinical AZ is not. We thus treated the mouse model with MTZ and cultured the VSMCs with AZ.

This study used aortic aneurysm and aortic dissection tissue samples to investigate the calcification mechanism in AS. Our clinical imaging data showed calcification in these samples. Many studies have demonstrated that most aortic aneurysms and aortic dissections are caused by AS and are related to vascular calcification (Lavall et al., 2012; Ladich et al., 2016).

## Materials and Methods

### Acquisition of Human AS Tissue

The tissue specimens were acquired from patients undergoing heart surgery at Shandong Provincial Qianfoshan Hospital Affiliated with Shandong University (Jinan, China). The control specimens were acquired from volunteers who served as heart transplant donors (*n* = 7), and information on the volunteers was confidential. AS tissue specimens were acquired from those patients with aortic aneurysm or aortic dissection accompanied by AS symptoms (*n* = 7). The patients had hypertension and aortic calcification, as shown by CT (Discovery 750, GE Healthcare, USA) imaging examination. Detailed information about the patients is shown in **Supplementary Table 1**. All aortic tissues were obtained from the ascending aortas. The study protocol was approved by the Medical Ethics Committee of Shandong Provincial Qianfoshan Hospital at Jinan (approval number: 20170607).

### Rat VSMC Culture and Calcification Induction

Rat VSMC culture and the induction of calcification were performed using previously described methods (Liu et al., 2016). VSMCs at the 3^rd^ to 8^th^ passages that grew well were used for subsequent calcification induction experiments. The cells were plated at a density of 1 × 10^5^ cells. Upon reaching 80% confluence, the cells were cultured in calcification medium that contained 10 mM β-glycerophosphate (β-GP) (Sigma, USA) for 14 days, and the medium was changed every 3 days. The experimental design was based on the study by Liu et al. (2010).

### Effects of AZ on Rat VSMC Calcification

The CA inhibitor AZ (Sigma, USA) was dissolved in 0.05% dimethylsulfoxide (DMSO). Rat VSMCs were plated at a density of 1 × 10^5^ cells per well, and 100 µmol/L AZ was added to the cells undergoing calcification induction. The experiments were performed as previously described (Hall and Kenny, 1985a; Hall and Kenny, 1985b). In the control culture, 0.05% DMSO was added to the calcification induction medium.

### Analysis of Alizarin Red S Staining

On day 14 after the induction of rat VSMC calcification, the cells were washed with phosphate buffer saline (PBS) (Solabio, China), fixed with 95% ethanol for 10 min, and stained with 0.5% (w/v) alizarin red S (AR-S, Solabio) (pH = 4.2) for 30 min at room temperature. The formation of calcified nodules in the VSMCs was examined under a microscope. Rat VSMCs treated with anti-CA1 siRNA were analyzed by the same protocol.

### Quantification of Calcification Using Cetylpyridinium Chloride

Following AR-S staining, VSMCs that underwent calcification induction were treated with 10% (w/v) cetylpyridinium chloride and incubated at 37°C for 1 h. The optical density (OD) was measured at a wavelength of 562 nm using a microplate reader (Molecular Devices, USA). VSMCs treated with anti-CA1 siRNA were analyzed by the same protocol.

### Examination of CA, Runx2, ALP, and BMP2 Expression Using Real-Time, Fluorescence-Based Quantitative PCR

Total RNA was extracted from the human tissues, cultured VSMCs, and mouse aortic tissues according to the RNApure Tissue & Cell kit instruction manual (CWbiotech, China). The total RNA was reverse transcribed into complementary deoxyribonucleic acid (cDNA) according to the instructions of a reverse transcription kit (Toyobo, Japan); messenger ribonucleic acid (mRNA) expression was then determined using real-time fluorescence-based quantitative PCR (StepOnePlus, Life Technology, USA). Glyceraldehyde-3-phosphate dehydrogenase (GAPDH) mRNA was used as an internal control to quantify expression of the target genes. Each sample was examined in triplicate. The mRNA expression relative to GAPDH was measured using the comparative 2^−ΔΔCt^ method (Livak and Schmittgen, 2001). The specificity of the primers was determined using melting curve analysis. The primer sequences are listed in **Supplementary Table 2**.

### Examination of CA1 Expression Using Western Blotting (WB)

The human tissue specimens, mouse aorta tissue specimens, and cultured VSMCs were homogenized in Radio-Immunoprecipitation Assay (RIPA) lysis buffer (Beyotime, China) on ice and centrifuged at 12,000 rpm. Proteins were separated using 12% sodium dodecyl sulfate–polyacrylamide gel electrophoresis (SDS-PAGE) and transferred to a PVDF membrane. The membrane was incubated with an anti-CA1 antibody (Abcam, USA, catalog number: 108367) at 4°C overnight. The membrane was then incubated with a horseradish peroxidase (HRP)-conjugated goat anti-rabbit secondary antibody at 37°C for 1 h. The membrane was developed using Western Chemiluminescent HRP Substrate (ECL, Millipore). The expression level of GAPDH was used as an internal control. The grayscale value of the target protein was quantified using ImageJ software (National Institutes of Health, USA).

### Transfection of Anti-CA1 siRNA

Anti-CA1 siRNA was synthesized by GenePharma (China), and the sequence was 5’-GGAUGCCCUAAGCUCAGUUTT-3’. The transfection was performed with PepMute^™^ siRNA Transfection Reagent (SignaGen, USA). Following transfection, the VSMCs were harvested, and proteins were extracted. An Allstars siRNA that does not suppress the expression of any gene was used as the control.

### Examination of VSMC Proliferation Using the Cell Counting Kit-8 (CCK-8) Assay

Rat VSMCs were harvested after transfection with anti-CA1 siRNA. Cell Counting Kit-8 solution (Dojindo, Japan) was added, and the cells were cultured for 2 h. The OD was measured at 450 nm using a microplate reader.

### Examination of VSMC Migration Using Transwell Chambers

After transfection with anti-CA1 siRNA, Transwell chambers (Corning, USA) were placed in 24-well plates. Dulbecco’s modified Eagle medium (DMEM) containing 10% fetal bovine serum (FBS) was added to the bottom chamber, and serum-free cell suspension (containing approximately 8 × 10^4^ cells) was added to the top chamber. The cells were cultured at 37°C for 24 h. The cells in the upper well of the chambers were completely removed with a cotton swab to ensure that they would not affect subsequent experiments. The chambers were fixed with methanol for 15 min, and the upper wells were wiped again after the chambers were dry. The chambers were then stained with 0.01% crystal violet, and the cells were observed and counted under a microscope.

### Flow Cytometry Analysis of VSMC Apoptosis Using Annexin V-FITC/Propidium Iodide (PI) Staining

VSMCs were transfected with anti-CA1 siRNA for 48 h, and 5–10 × 10^4^ cells from each group were centrifuged, washed with PBS, resuspended in binding buffer, and stained with Annexin V-FITC and PI (Dakewei, China) in the dark for 15 min. The cells were examined using flow cytometry (NovoCyte D2040R, USA).

### Establishment of an AS Mouse Model

Eight-week-old healthy male C57BL/6J-ApoE^−/−^ mice weighing 22 ± 2 g were purchased from Vital River Laboratory Animal Technology in Beijing, China. The mice were randomly divided into four groups: 1) Control group (*n* = 40): ApoE^−/−^ mice were fed a normal diet for 21 weeks; from weeks 1 to 21, the mice were administered normal saline at a dose of 0.1 ml/10 g body weight by oral gavage every other day; 2) AS model group (*n* = 40): ApoE^−/−^ mice were fed a high-fat diet (1% cholesterol and 10% fat in the regular diet) for 21 weeks; from week 1 to 21, the mice were administered normal saline at a dose of 0.1 ml/10 g body weight by oral gavage every other day; 3) MTZ treatment group (*n* = 40): ApoE^−/−^ mice were fed a high-fat diet for 21 weeks; from weeks 1 to 12, the mice were administered normal saline at a dose of 0.1 ml/10 g body weight by oral gavage every other day, and from weeks 12 to 21, the mice were administered MTZ at a dose of 25 mg/kg body weight/day by oral gavage every other day (Wang et al., 2009; Konstantopoulos et al., 2012); and 4) MTZ preventive treatment group (*n* = 40): ApoE^−/−^ mice were fed a high-fat diet for 21 weeks; from weeks 1 to 21, the mice were administered MTZ at a dose of 25 mg/kg body weight/day by oral gavage every other day, and at week 21, all mice were humanely euthanized by a lethal dose of ketamine and xylazine, and the aorta was isolated. The study protocol was approved by the Medical Ethics Committee of Shandong Provincial Qianfoshan Hospital at Jinan (approval number: 20170607). The breeding and handling of the experimental animals were carried out in accordance with the Helsinki Convention on Animal Protection and the Regulations of the People’s Republic of China on the Administration of Experimental Animals.

### Measurement of Mouse Blood Lipid Levels

Blood was collected and centrifuged at 3,000 rpm for 10 min. The mouse serum levels of total cholesterol (TC), triglycerides (TG), low-density lipoprotein cholesterol (LDL-c), and high-density lipoprotein cholesterol (HDL-c) were measured using a kit from Nanjing Jiancheng Bioengineering Institute (China), and the atherogenic index (AI) was calculated as follows: AI = [TC − (HDL-c)]/HDL-c.

### Measurement of Mouse Serum Nitric Oxide (NO) Level

Blood serum was collected from mice in the four groups, and the NO level was measured using a kit from Nanjing Jiancheng Bioengineering Institute (China).

### Measurement of Inflammatory Cytokines in VSMC Culture and Mouse Serum

The VSMC culture medium was collected after culturing, and the serum from mice was collected at week 21. The levels of inflammatory cytokines were examined by flow cytometry (NovoCyte D2040R, USA) using an inflammatory cytokine kit. The VSMC culture medium was examined using the Rat Th1/Th2 Panel with filter plate (BioLegend, USA); the serum from mice was examined using the Mouse Inflammation Panel with filter plate (BioLegend, USA). The capture bead mixture, fluorescence reagent, and the sample were added to the detection plate wells. The plate was then placed on a shaker and incubated in the dark at room temperature for 2 h. After two washes, the mixture was resuspended in wash buffer. The changes in the levels of IL-1α, IL-1β, IL-2, IL-4, IL-5, IL-6, IL-10, IL-12p70, IL-13, IL-17A, IL-18, IL-33, interferon (IFN)-γ, granulocyte-macrophage colony-stimulating factor (GM-CSF), tumor necrosis factor-α (TNF-α), chemokine (C-X-C motif) ligand 1/keratinocyte-derived chemokine (CXCL1/KC), and C-C motif chemokine ligand 2 (CCL2)/monocyte chemoattractant protein 1 (MCP-1) were analyzed using FCAP_Array_v3 software (BD Biosciences, USA).

### Observation of Mouse Atherosclerotic Plaques Using Sudan IV Staining

The thoracoabdominal aorta was carefully isolated up to the iliac artery branch. The aorta was placed in Sudan IV solution for 20 min and differentiated in 80% ethanol for 20 min. The aorta was observed and imaged under a microscope. The extension of atherosclerotic plaques was semiquantified by calculating the ratio of the red-stained area in aorta to the total aortic area using Image-Pro Plus 6.0 (Media Cybernetics, USA) (Zeng et al., 2014).

### Observation of Mouse AS Plaques Using Oil Red O Staining

Cryostat sections of the aortas from each mouse group were prepared, incubated with 70% ethanol, and stained with freshly prepared Oil Red O working solution for 10 min. The sections were washed in 70% ethanol followed by distilled water and then counterstained with hematoxylin and placed under running water for bluing. The sections were then mounted with glycerol jelly mounting medium. The atherosclerotic plaques were observed and imaged under a light microscope. The extent of the aortic root atherosclerotic plaques was semiquantified by calculating the Oil Red O-positive staining area in the sections using ImageJ software (Kulathunga et al., 2018; Li et al., 2019).

### Hematoxylin and Eosin (HE) Staining of Mouse Aorta Tissue

Mouse aorta tissue samples were fixed with 4% paraformaldehyde, embedded in paraffin, and sectioned continuously. After deparaffinization and dehydration, the sections were stained with HE.

### Observation of Calcium Deposition in Human and Mouse Aortas Using von Kossa Staining

A tissue array containing AS human aortic tissue samples (*n* = 8) and normal human aortic tissue samples (*n* = 8) commercially obtained from Alenabio (China) was used for von Kossa staining. Detailed information on the tissue array is provided in **Supplementary Table 3**. The mouse aortic tissues were dehydrated, embedded, and sliced into 5 µm thick sections. After routine deparaffinization and dehydration, the sections were incubated with a von Kossa (Solabio, China) silver solution under bright light for 15 min and then treated with a hypotonic solution (sodium thiosulfate) for 2 min. The nuclei were counterstained with HE. Sections were then dehydrated, cleared, and mounted with neutral balsam. The status of calcium deposition in the mouse aorta was observed and imaged under a light microscope.

### Immunohistochemistry of CA1 Expression

Paraffin sections of mouse aorta and human aorta were deparaffinized. The tissue array (Alenabio, China) used for immunohistochemistry of human tissue contained continuous tissue sections for von Kossa staining. The sections were incubated with an anti-CA1 polyclonal antibody (Cusabio, China) overnight at 4°C. The sections were then incubated with goat anti-rabbit IgG (Zhongshan Golden Bridge Biotechnology, China). Sections were treated with diaminobenzidine (DAB, Zhongshan Golden Bridge Biotechnology, China) and counterstained with hematoxylin.

### Statistical Analysis

Normality and homogeneity of variance tests were performed using SPSS 17.0 software (IBM, USA). Data that met the test criteria were represented as the means ± SEM. The significance of differences among multiple groups was analyzed using one-way analysis of variance (ANOVA). Comparisons between groups were analyzed using Fisher’s least significant difference (LSD) method. Paired and/or unpaired Student’s *t*-tests were used to evaluate the statistical significance of differences between two groups. *P* < 0.05 was considered to be statistically significant.

## Results

### CA1 Expression in Human Aortic Tissue

**Figure 1:**
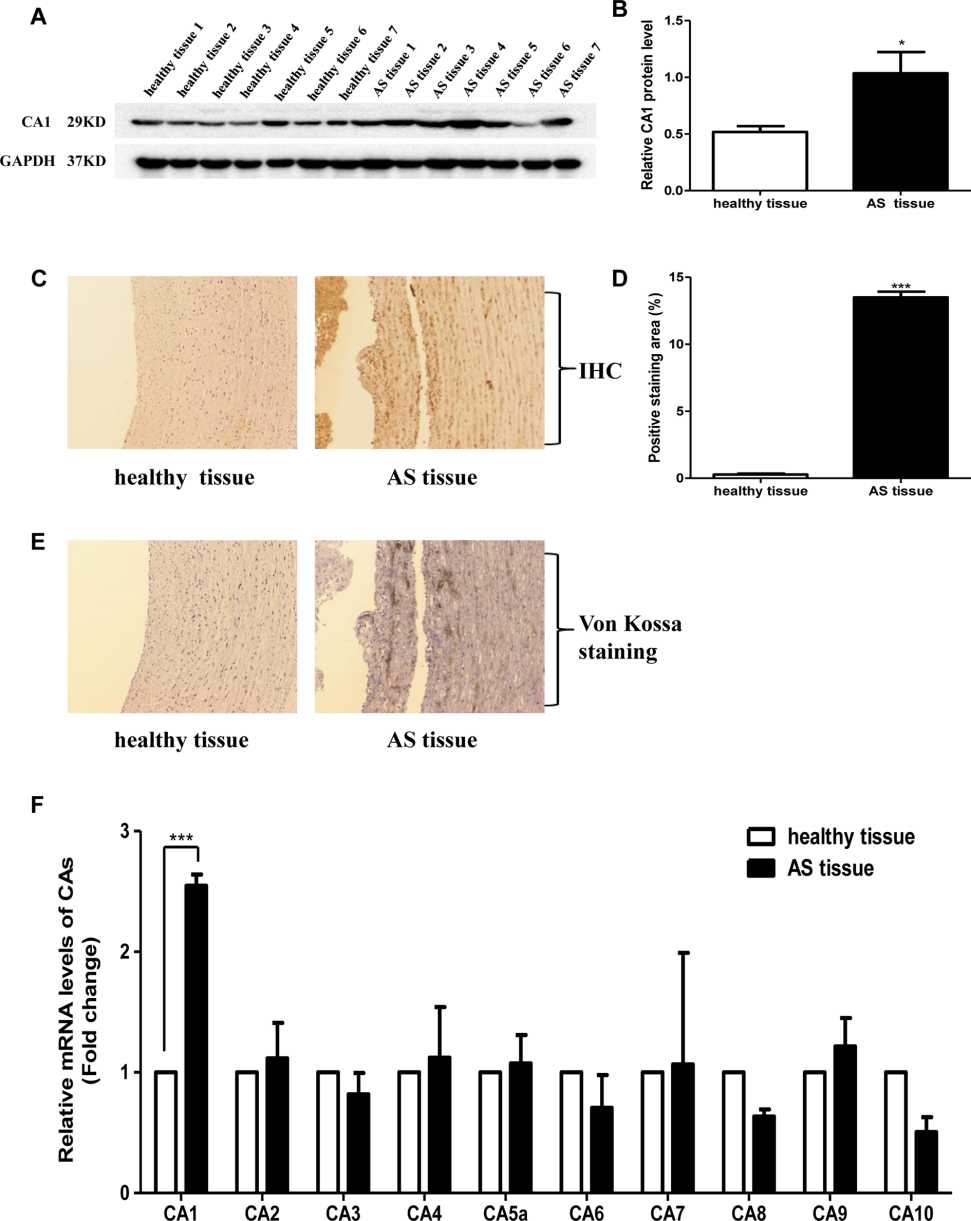
Carbonic anhydrase 1 (CA1) expression and calcification in human aortic tissues. **(A)** CA1 protein expression in human atherosclerosis (AS) tissues and healthy aortic tissues using Western blot analysis. **(B)** Semiquantification of CA1 protein expression in human aortic tissues. **(C)** CA1 protein expression in human aortic tissues using immunohistochemistry (100X magnification). **(D)** Semiquantification of CA1-positive staining in human aortic tissues. **(E)** Calcification in human aortic tissues using von Kossa staining (100X magnification). **(F)** The expression levels of each CA member in human AS aortic tissues and healthy aortic tissues determined using real-time PCR. Data are shown as the means ± SEM. *P < 0.05, ***P < 0.001.

The expression of CA1 in human ascending aortic tissues was evaluated. Western blot analysis showed significantly increased expression of CA1 protein at a molecular weight of 29 kD in the human AS tissues compared with that in the healthy aortic tissues (*P* = 0.0204, **Figures 1A, B**). Immunohistochemistry also revealed CA1 expression in human aortic AS tissues. Many cells in the vascular wall were immunostained with anti-CA1 antibody in the diseased tissues. However, little CA1 immunosignal was observed in the healthy samples (*P* < 0.001, **Figures 1C, D**). Additionally, von Kossa staining revealed obvious dark brown calcium deposition in continuous tissue sections of the human aortic AS samples. No identifiable calcium deposition was found in the healthy samples (**Figure 1E**). mRNA levels of CA1, CA2, CA3, CA4, CA5a, CA6, CA7, CA8, CA9, and CA10 were examined in the aortic aneurysm tissues, aortic dissection tissues, and healthy aortic tissues using real-time PCR. Only CA1 exhibited significantly increased expression in the AS aortic samples (*P* < 0.001, **Figure 1F**). The above results demonstrated that the expression of CA1 and calcium salt deposition was increased in human aortic AS tissues.

### The Effects of MTZ on Mouse AS

The AS mouse model was established by feeding the ApoE^−/−^ mice a high-fat diet. Compared to the control group, the body weights of the mice in all three other groups increased more rapidly. However, no significant difference was observed among the three groups that were fed the high-fat diet (**Supplementary Figure 1**).

**Figure 2:**
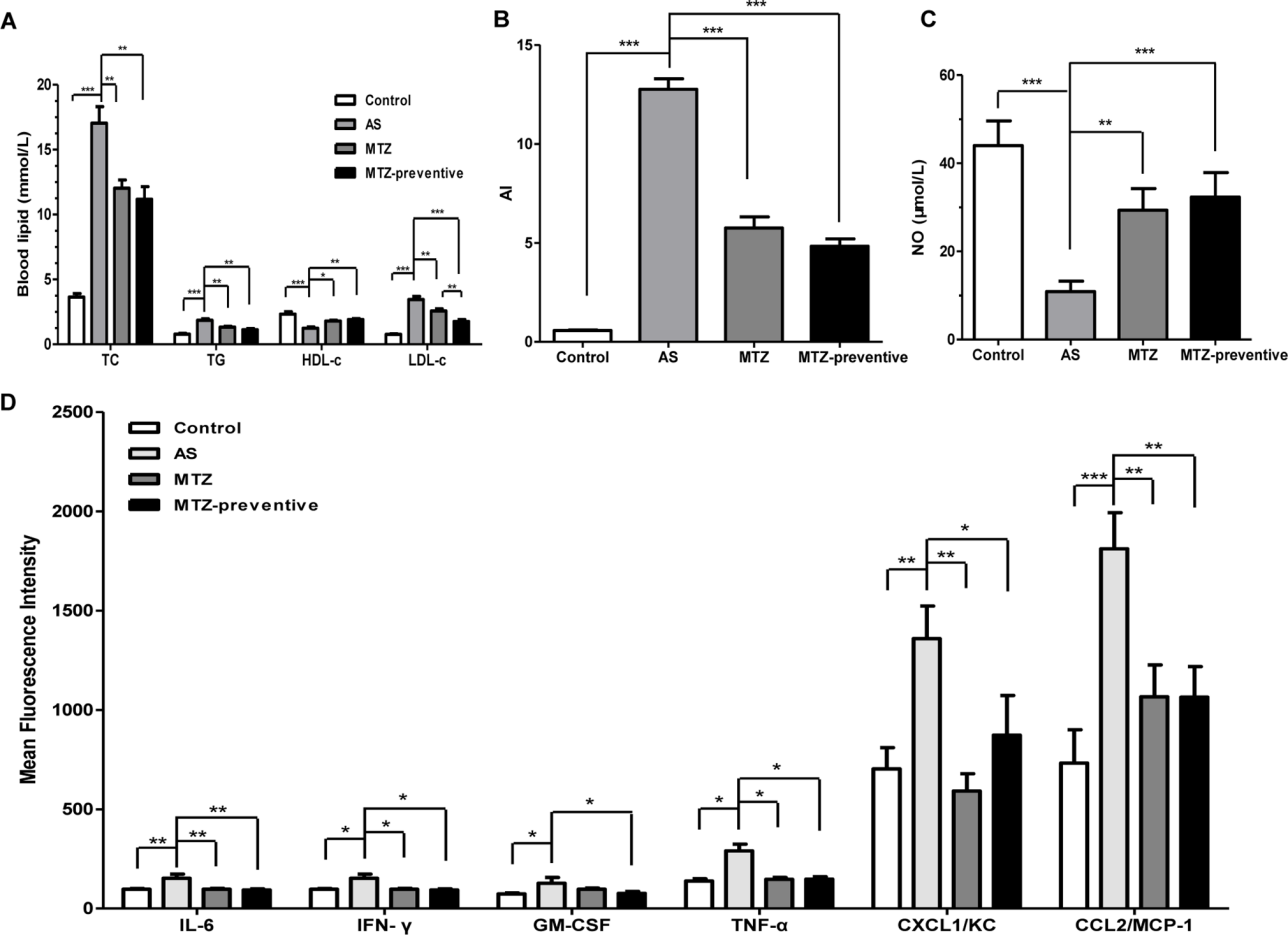
Changes in serological AS indicators in mice treated with methazolamide (MTZ). Serum levels of **(A)** total cholesterol (TC), triglycerides (TG), low-density lipoprotein cholesterol (LDL-c), and high-density lipoprotein cholesterol (HDL-c) (ANOVA, *P* < 0.001, < 0.001, < 0.001, and 0.002, respectively). **(B)** atherogenic indexes (AIs) (ANOVA, *P* < 0.001). **(C)** Nitric oxide (NO) (ANOVA, *P* < 0.001). **(D)** Proinflammatory cytokines in the four groups of mice (ANOVA, *P* = 0.004, 0.028, 0.004, < 0.001, < 0.001, and 0.002, respectively). *N* = 10 for each group. Data are shown as the means ± SEM. *P < 0.05, **P < 0.01, ***P < 0.001.

After 21 weeks, blood was collected from the mice in all four groups for serum lipid analysis. Compared to the healthy control group, the AS model mice exhibited significantly higher serum TC, TG, and LDL-c levels (*P* < 0.001, < 0.001, and 0.001, respectively) and significantly lower HDL-c levels (*P* < 0.001), indicating successful establishment of the AS animal model. Compared to the AS model group, the MTZ treatment significantly reduced serum TC, TG, and LDL-c levels (*P* = 0.0036, 0.0039, and 0.0056, respectively) and increased HDL-c levels (*P* = 0.012) in the mice; the mice in the MTZ preventive treatment group also displayed significantly reduced serum TC, TG, and LDL-c levels (*P* = 0.0014, 0.0013, and <0.001, respectively) and increased HDL-c levels (*P* = 0.004). The difference in the levels of TC, TG, and HDL-c between the MTZ treatment group and the MTZ preventive treatment group was not statistically significant (*P* = 0.502, 0.158, and 0.506, respectively), but the difference in the LDL-c levels was statistically significant (*P* = 0.009) (**Figure 2A**). The AI of the AS model group was significantly elevated (*P* < 0.001). Compared to the AS model group, the AIs of the MTZ treatment and preventive treatment groups were significantly reduced (both *P* values were <0.001, **Figure 2B**). Measurement of the serum NO levels in the mice from each group showed that the serum NO level in the AS model was significantly reduced compared to that of the healthy controls (*P* < 0.001). Compared to the AS model group, the serum NO levels in both the MTZ treatment group and the preventive treatment group were significantly increased (*P* = 0.002 and *P* < 0.001, respectively). Though the serum NO levels in the preventive treatment group were increased, there was no significant difference between the MTZ treatment group and the MTZ preventive group (**Figure 2C**). The above results demonstrated that MTZ had a significant therapeutic effect on AS in the animal model.

The levels of different cytokines were assessed using flow cytometry. Compared to the control group, the levels of IL-6, IFN-γ, GM-CSF, TNF-α, CXCL1/KC, and CCL2/MCP-1 were significantly increased in mice from the AS model group (*P* = 0.0026, 0.015, 0.037, 0.014, 0.004, and <0.001, respectively), but the levels of the other analyzed cytokines (IL-1α, IL-1β, IL-2, IL-4, IL-5, IL-10, IL-12p70, IL-13, IL-17A, IL-18, and IL-33) were not significantly different between the two groups. Compared to the AS model group, the levels of IL-6, IFN-γ, TNF-α, CXCL1/KC, and CCL2/MCP-1 were significantly reduced in mice from the MTZ treatment group (*P* = 0.0015, 0.036, 0.02, 0.00105, and 0.005, respectively). Meanwhile, the levels of IL-6, IFN-γ, TNF-α, GM-CSF, CXCL1/KC, and CCL2/MCP-1 were also significantly reduced in mice from the MTZ preventive treatment group (*P* = 0.0027, 0.037, 0.046, 0.019, 0.0296, and 0.007, respectively). Compared to the MTZ treatment group, the levels of these inflammatory cytokines in the MTZ preventive treatment group did not change significantly (*P* = 0.208, 0.076, 0.215, 0.850, 0.170, and 0.996, respectively; **Figure 2D**). The above results demonstrated that MTZ suppressed the production of some inflammatory cytokines in the AS model.

**Figure 3:**
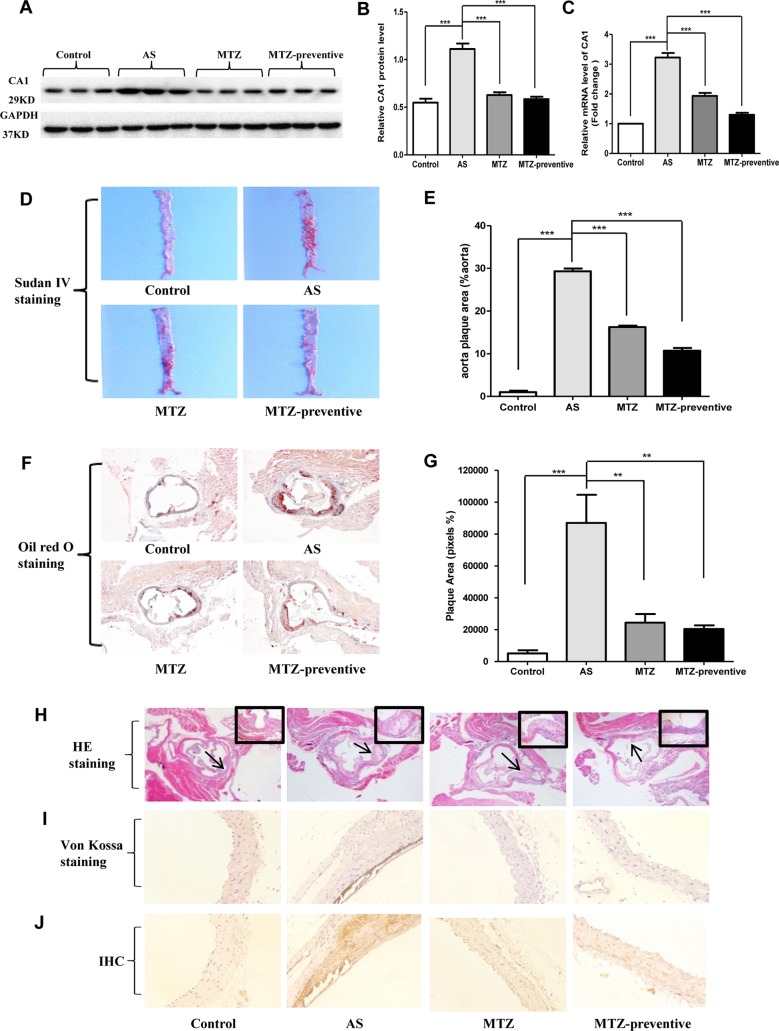
Changes in histopathological structure, calcification, and CA1 expression in aortas from AS mice treated with MTZ. **(A)** CA1 protein expression in mouse model aortas using Western blot analysis. **(B)** Semiquantification of CA1 protein expression in mouse model aortas (ANOVA, *P* < 0.001). **(C)** CA1 transcription in aortas from the mice using real-time PCR (ANOVA, *P* < 0.001). **(D)** Sudan IV staining. **(E)** Semiquantification of atherosclerotic lesion area of AS mice (ANOVA, *P* < 0.001). **(F)** Oil Red O staining (40X magnification). **(G)** Semiquantification of atherosclerotic lesion area of the aortic root (ANOVA, *P* < 0.001). **(H)** Hematoxylin and eosin (HE) staining (40X magnification, the area indicated by the arrow is shown in the top right corner at 200X magnification). **(I)** Von Kossa staining (200X magnification). **(J)** Immunohistochemistry of CA1 (200X magnification). *N* = 5 for each group. Data are shown as the means ± SEM. **P < 0.01, ***P < 0.001.

CA1 expression levels in aortic tissue samples from the AS mouse model were examined using Western blot and real-time PCR. Compared with the control group, CA1 protein with a molecular weight of 29 kD showed significantly higher expression levels in the AS mice (*P* < 0.001). Compared with the AS model group, the CA1 protein level was significantly reduced in the treatment group and in the preventive treatment group (*P* < 0.001 and *P* < 0.001, respectively; **Figures 3A, B**). The CA1 mRNA level was also significantly increased in the AS model aortic tissue and was decreased in the MTZ treatment group and MTZ preventive treatment group (*P* < 0.001 and *P* < 0.001, respectively; **Figure 3C**). The levels of other CA members in the aortic tissue samples from the AS animal model were also examined using real-time PCR. Compared with the expression profile of the healthy controls, mRNA levels of CA9 and CA10 in addition to CA1 were increased in the AS group (*P* = 0.029 and *P* = 0.025, respectively), but the transcription levels of other CA members from CA2 to CA8 did not significantly change in the animals. Furthermore, all CA members except CA1 had no significant change in their mRNA levels in the MTZ treatment and MTZ preventive treatment groups (**Supplementary Figure 2**). The above results demonstrated that CA1 had increased expression in aortic AS tissues and that MTZ treatment principally reduced CA1 expression in the tissues.

At week 21 of feeding, mouse aorta samples were stained with Sudan IV. Compared to the control group, the area of the AS plaques in the aorta of the AS model group was significantly increased (*P* < 0.001), while compared to the AS model group, this value was decreased in the MTZ treatment group (*P* < 0.001); the AS plaque area was even smaller in the aortas of the MTZ preventive treatment group (*P* < 0.001, **Figures 3D, E**). The mouse aorta samples were also examined using Oil Red O staining. The aorta lumen in the control group was smooth, and few AS plaques were observed. The most striking red staining was observed in the aorta intima of the AS model group, and massive fat dots, stripes, and typical AS plaques protruded from the aorta intima. AS plaques were also observed in the MTZ treatment and preventive treatment groups but to a lesser extent than those in the AS model group (*P* = 0.0015 and *P* = 0.00102, respectively; **Figures 3F, G**). HE staining did not show plaques in the aortas of mice from the control group. The internal vascular wall was thin and small, the endothelial cells and VSMCs were aligned in order, and the media thickness was normal in the healthy controls. In the aortas of the AS model group, clear concentric AS plaques, accompanied by massive foam cells that accumulated inside the plaques, were observed. Additionally, large amounts of cholesterol crystals were present. The smooth muscle layer was relaxed and structurally disrupted, and inflammatory cells had accumulated. The intima was thickened and protruded into the lumen, leading to stenosis of the lumen. However, those pathological changes were obviously alleviated in the MTZ treatment and preventive treatment groups (**Figure 3H**). Thus, the administration of MTZ to ApoE^−/−^ mice by oral gavage significantly alleviated AS pathogenesis.

Von Kossa staining was used to detect calcium deposition in the mouse aorta. The assay showed a large amount of dark brown calcium deposition in the intima of the AS model mice. Compared to the AS model group, identifiable calcium deposition was not observed in the MTZ treatment and preventive treatment groups, indicating that the MTZ treatment significantly suppressed calcification in AS mice (**Figure 3I**). CA1 expression in the mouse aortic tissue sections was examined using immunohistochemistry. Compared to the control group, a large amount of brown immunoreactive signal was present in the AS model group, indicating that CA1 was expressed at high levels in the AS mice. Furthermore, the CA1 expression was localized in the calcified regions by comparing the successive sections with von Kossa staining. Compared to the AS model group, the immunoreactivity in the MTZ treatment group was significantly reduced, suggesting that the alleviation of AS was accompanied by reduced CA1 expression. Similarly, the level of immunoreactivity in the preventive treatment group was also significantly less than that in the AS model group, indicating a reduction in CA1 expression (**Figure 3J**). The above observations demonstrated that CA1 was extensively expressed in the AS model and that MTZ suppressed the expression. Furthermore, the CA1 expression was colocated with calcium deposition, and MTZ inhibited calcification in the aortic tissue of AS mice.

### Effect of CA1 Expression and AZ on Rat VSMC Calcification

**Figure 4:**
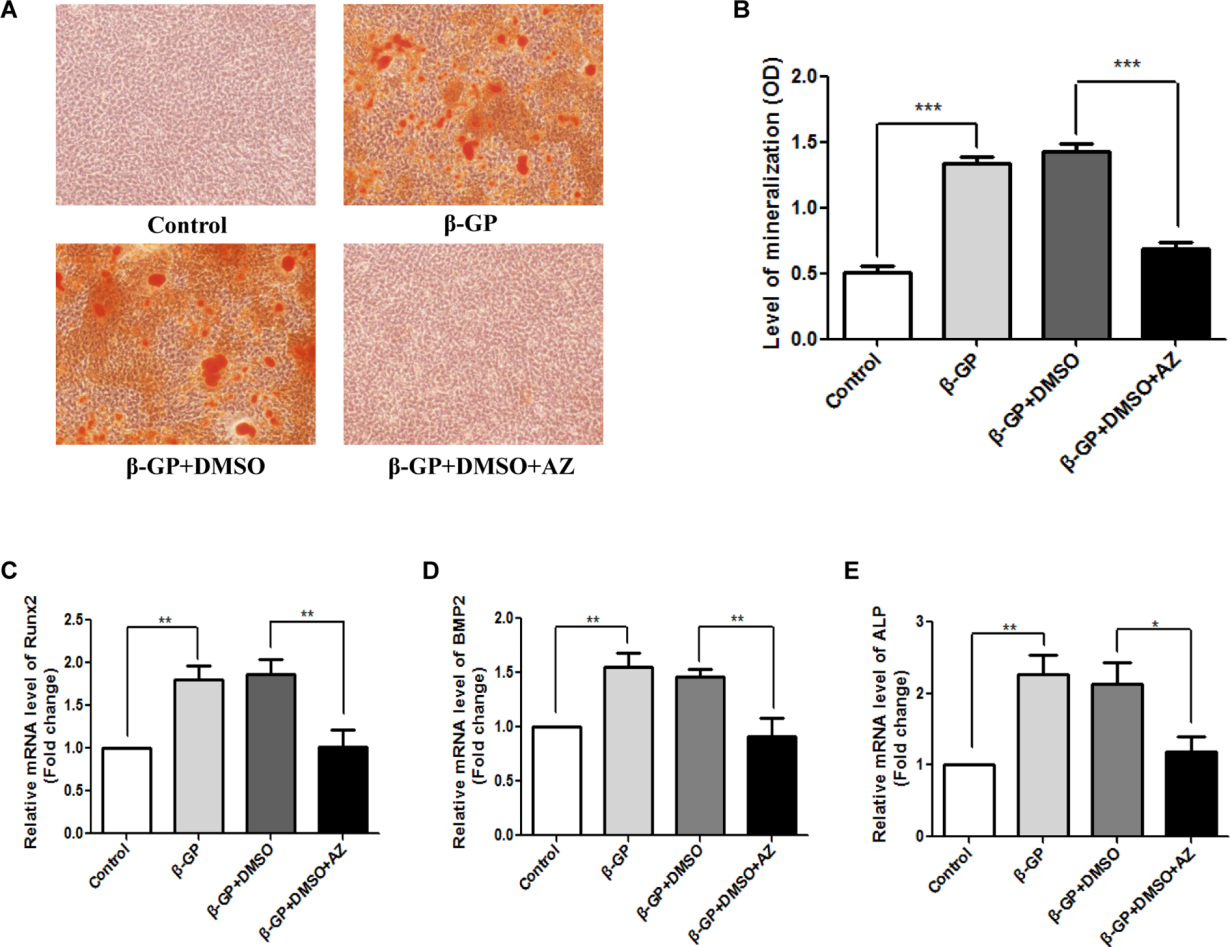
Calcification in rat vascular smooth muscle cells (VSMCs) with acetazolamide (AZ) treatment. **(A)** VSMC calcification using alizarin red S (AR-S) staining. **(B)** Quantification of calcification using cetylpyridinium chloride (ANOVA, *P* < 0.001). Runt-related transcription factor 2 (Runx2) **(C)**, alkaline phosphatase (ALP) **(D)**, and bone morphogenetic protein 2 BMP2 **(E)** mRNA expression in rat VSMCs using real-time PCR (ANOVA, *P* < 0.001, 0.003, and 0.003, respectively). The results were derived from three independent experiments. Data are shown as the means ± SEM. *P < 0.05, **P < 0.01, ***P < 0.001.

Rat VSMC calcification was induced with β-GP, and the cells were simultaneously treated with the CA1 inhibitor AZ. Because AZ was dissolved in DMSO, we also added DMSO to the culture as a control. AR-S staining showed a large amount of orange, calcified nodules in the rat VSMCs. No calcified nodules were observed in cells that did not receive β-GP treatment, and few calcified nodules were observed in cells treated with AZ (**Figure 4A**). Quantitative analysis with cetylpyridinium chloride showed a similar increase in cellular calcification upon β-GP induction (*P* < 0.001). The AZ treatment drastically reduced calcification (*P* < 0.001) to a level similar to that of cells without calcification induction (*P* > 0.05, **Figure 4B**). The mRNA expression of the ossification markers Runx2, ALP, and BMP2 in the rat VSMCs was analyzed before and after calcification using real-time PCR. Compared to the control group, the induction of calcification significantly increased the expression of Runx2, ALP, and BMP2 (*P* = 0.006, 0.008, and 0.004, respectively), indicating that the rat VSMCs underwent a biomineralization process upon calcification induction. The AZ treatment significantly suppressed Runx2, ALP, and BMP2 expression in VSMCs (*P* = 0.005, 0.008, and 0.017, respectively; **Figures 4C–E**). The above results demonstrated that AZ inhibited the calcification of VSMCs.

**Figure 5:**
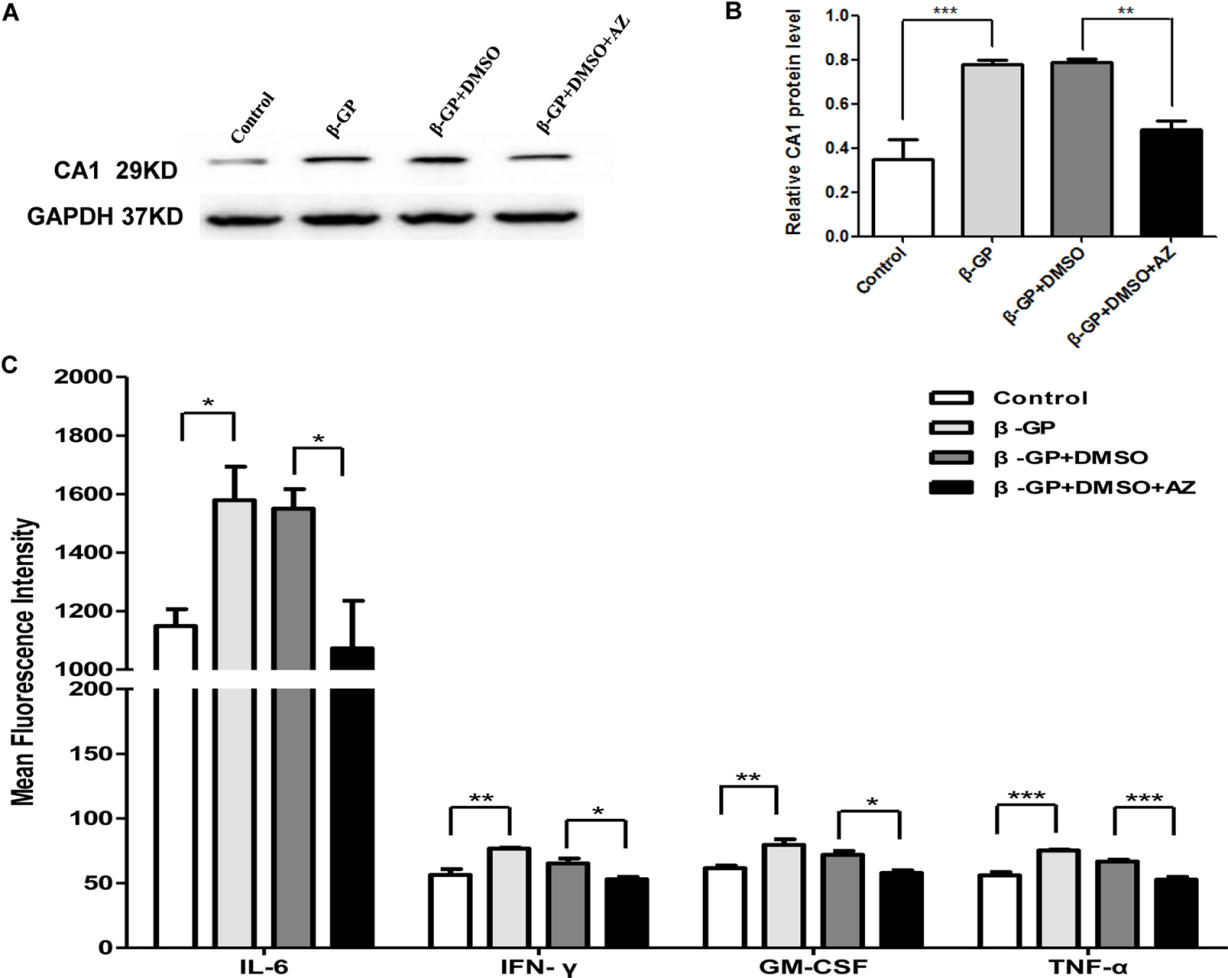
Expression of CA1 protein and inflammatory cytokines in rat VSMCs with AZ treatment. **(A)** Western blot analysis of CA1 protein expression in rat VSMCs. **(B)** Semiquantification of CA1 protein expression (ANOVA, *P* < 0.001). **(C)** Cytokine levels in supernatant of cultured VSMCs by flow cytometry (ANOVA, *P* = 0.020, 0.003, 0.004, and <0.001, respectively). The results were obtained from three independent experiments. Data are shown as the means ± SEM. *P < 0.05, **P < 0.01, ***P < 0.001.

The CA1 protein level in rat VSMCs was analyzed using WB. The induction of calcification significantly increased CA1 protein levels in rat VSMCs (*P* < 0.001), and the AZ treatment significantly suppressed CA1 expression (*P* = 0.003, **Figures 5A, B**). These results confirmed that CA1 expression was elevated during the cellular calcification. Compared to the control group, the levels of IL-6, IFN-γ, GM-CSF, and TNF-α in rat VSMC suspension were significantly increased after induction of calcification (*P* = 0.023, 0.002, 0.003, and <0.001, respectively). The AZ treatment significantly reduced the levels of IL-6, IFN-γ, GM-CSF, and TNF-α (*P* = 0.014, 0.026, 0.011, and <0.001, respectively; **Figure 5C**). The levels of the other analyzed cytokines (IL-2, IL-4, IL-5, IL-10, and IL-13) were not significantly changed in the culture that received AZ treatment. The above results demonstrated that CA1 expression in VSMCs was accompanied by calcification and AZ suppressed this expression. AZ also inhibited inflammatory cytokine production during VSMC calcification.

**Figure 6:**
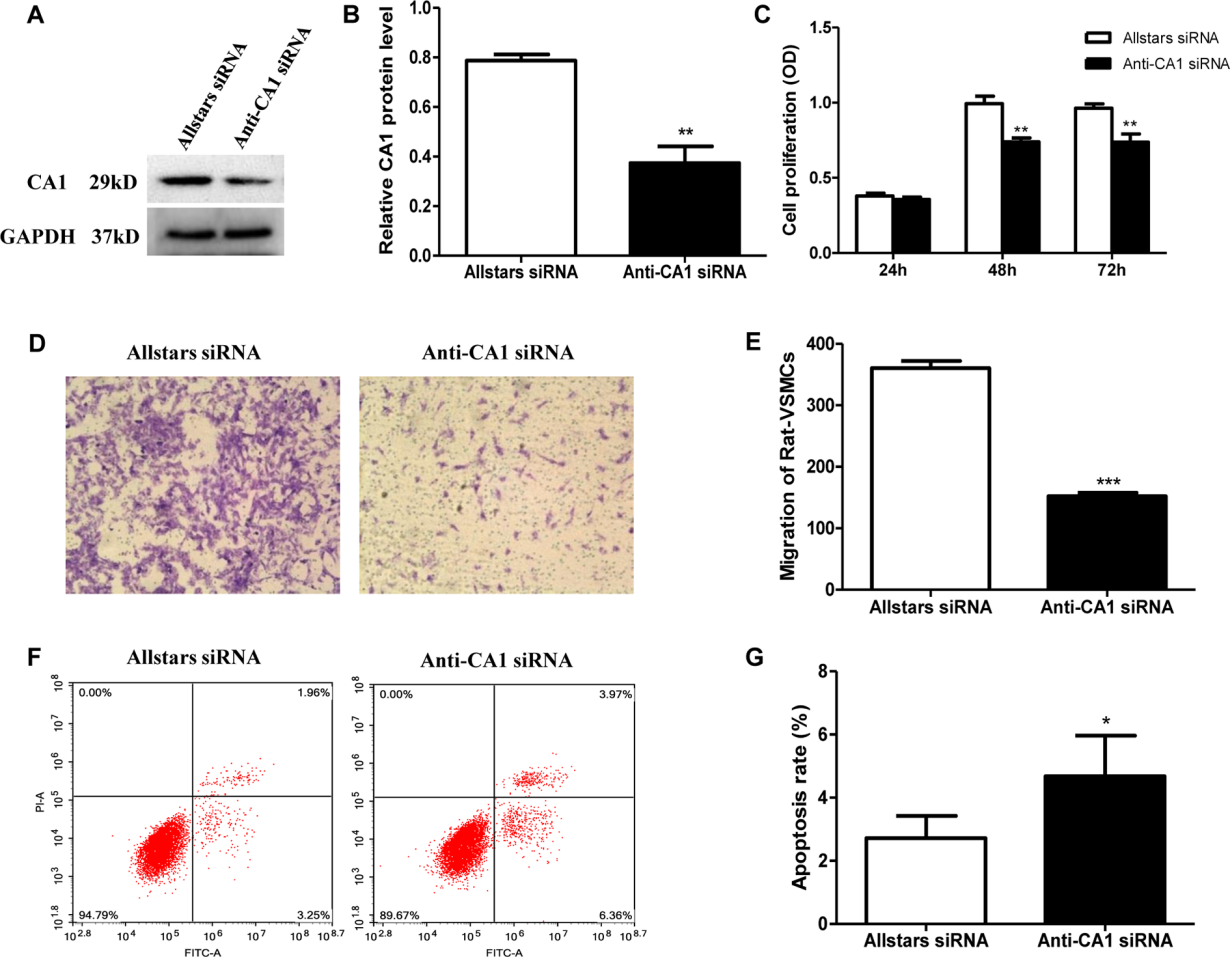
Rat VSMC proliferation, migration, and apoptosis following anti-CA1 siRNA transfection. **(A)** Western blot analysis of CA1 protein expression in rat VSMCs transfected by anti-CA1 siRNA. **(B)** Semiquantification of CA1 protein expression relative to the internal control. **(C)** Rat VSMC proliferation using the Cell Counting Kit-8 (CCK-8) assay. **(D)** Transwell migration of rat VSMCs. **(E)** Statistical analysis of cell migration. **(F)** Representative image of rat VSMC apoptosis by flow cytometry. **(G)** Quantification of the apoptotic cell ratio. The results were obtained from three independent experiments. Data are shown as the means ± SEM. *P < 0.05, **P < 0.01, ***P < 0.001.

Rat VSMCs were transfected with anti-CA1 siRNA. Transfection with Allstars siRNA was used as a control. Compared with the cells with Allstars siRNA, the anti-CA1 siRNA treatment significantly reduced the CA1 expression level by 53%, indicating effective inhibition of anti-CA1 siRNA on CA1 expression in cultured VSMCs (*P* = 0.0046, **Figures 6A, B**). Following anti-CA1 siRNA transfection, the cell proliferation of rat VSMCs was significantly decreased compared with that of the cells transfected with Allstars siRNA (*P* = 0.0021 and 0.0067, respectively; **Figure 6C**), and the cell migration of VSMCs with anti-CA1 siRNA transfection was also significantly inhibited (*P* < 0.001, **Figures 6D, E**). Anti-CA1 siRNA induced a significant increase in the apoptosis of rat VSMCs compared with that of the cells with Allstars siRNA transfection (*P* = 0.0353, **Figures 6F, G**).

**Figure 7:**
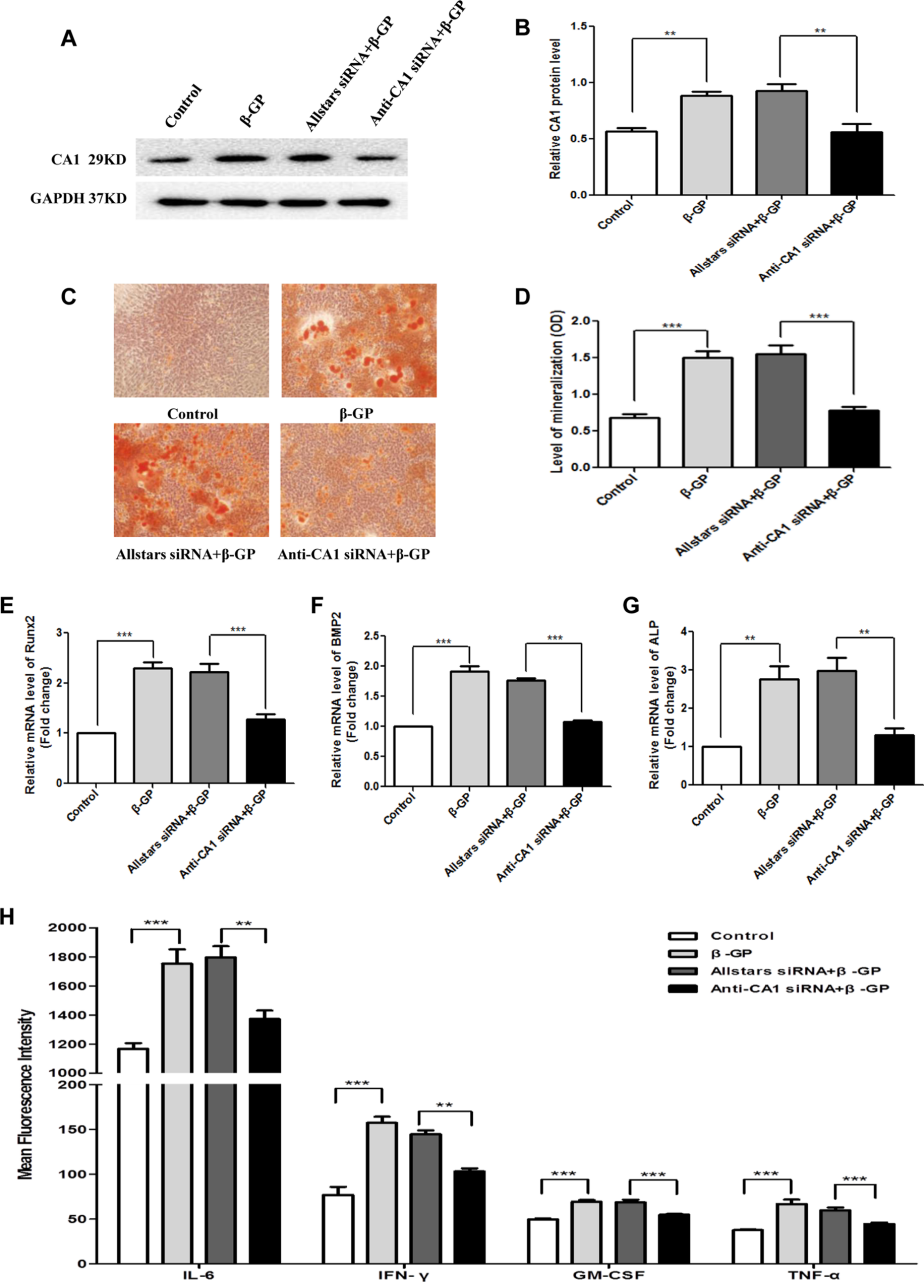
CA1 expression, calcification, and inflammatory cytokine production in rat VSMCs with anti-CA1 siRNA transfection. **(A)** Western blot analysis of CA1 protein expression in rat VSMCs. **(B)** Semiquantification of CA1 protein expression (ANOVA, *P* < 0.001). **(C)** Calcification of rat VSMCs transfected with siRNA using AR-S staining. **(D)** Quantification of calcification using cetylpyridinium chloride (ANOVA, *P* < 0.001). Runx2 **(E)**, ALP **(F)**, and BMP2 **(G)** mRNA expression in rat VSMCs using real-time PCR (ANOVA, *P* < 0.001, < 0.001, and <0.001, respectively). **(H)** Cytokine levels in the supernatant of cultured VSMCs using flow cytometry (ANOVA, *P* < 0.001, < 0.001, < 0.001, and <0.001, respectively). The results were obtained from three independent experiments. Data are shown as the means ± SEM. **P < 0.01 and ***P < 0.001.

Rat VSMCs were transfected with anti-CA1 siRNA in the presence of β-GP. The CA1 protein levels in rat VSMCs with siRNA transfection were measured using WB analysis. Compared with the cells with Allstars siRNA transfection, anti-CA1 siRNA transfection significantly reduced the CA1 protein expression, indicating siRNA inhibition of CA1 expression (*P* = 0.0013, **Figures 7A, B**). Compared with VSMCs treated with β-GP and Allstars siRNA, the AR-S staining revealed fewer calcified nodules in the cells that were transfected with anti-CA1 siRNA in the presence of β-GP (**Figure 7C**). Quantitative analysis with cetylpyridinium chloride also showed a significant decrease in VSMC cellular calcification upon β-GP induction when the cells were transfected with anti-CA1 siRNA (*P* < 0.001, **Figure 7D**). Following anti-CA1 siRNA treatment, the Runx2, ALP, and BMP2 transcription in rat VSMCs was significantly lower than that of cells that received Allstars siRNA and β-GP treatment (*P* < 0.001, < 0.001, and 0.002, respectively; **Figures 7E–G**). These results demonstrated that anti-CA1 siRNA inhibited the calcification of VSMCs and that CA1 expression mediated cell calcification.

The change in cytokine production in VSMC supernatant was also measured using flow cytometry. Compared with the control cells transfected with Allstars siRNA in the presence of β-GP, the cells transfected with anti-CA1 siRNA had significantly reduced levels of IL-6, IFN-γ, GM-CSF, and TNF-α (*P* = 0.003, 0.002, < 0.001, and 0.007, respectively; **Figure 7H**). Meanwhile, the levels of the other analyzed cytokines (IL-2, IL-4, IL-5, IL-10, and IL-13) were not significantly changed following the treatment.

## Discussion

Calcification is a marker of AS and is used to predict AS severity (Rodondi et al., 2007). In the present study, CA1 protein was expressed at high levels in AS tissues of both aortic aneurysm and aortic dissection, with extensive calcification. This study also detected high CA1 expression levels in mouse AS aortas, accompanied by rich calcium deposits. The formation of AS plaques results from the influences of several types of cells in the vascular wall, including vascular endothelial cells, lymphocytes, monocytes/macrophages, and VSMCs (Bennett et al., 2016). VSMCs account for approximately 70% of all AS cells (Gabunia et al., 2017). In this study, the induction of rat VSMCs with β-GP led to massive calcium deposition, a significantly increased expression of ossification-related genes, including ALP, Runx2, and BMP2, and increased CA1 expression levels. Furthermore, treatment of rat VSMCs with the CA inhibitor AZ significantly suppressed CA1, ALP, Runx2, and BMP2 expression and inhibited cellular calcification. Additionally, anti-CA1 siRNA treatment decreased VSMC calcification (induced with β-GP) and suppressed CA1, ALP, Runx2, and BMP2 expression. We also examined the expression levels of CA1 through CA10 in human and mouse AS aortic tissues. Only CA1, CA9, and CA10 had significantly increased expression levels in the animal AS tissues, and only CA1 had increased expression in human AS tissues. Furthermore, MTZ treatment inhibited only CA1 expression and did not significantly suppress CA9 or CA10 expression in the animal model. Thus, these results suggest that the increased expression of CA1 is related to vascular calcification and osteoblastic transformation of VSMCs. MTZ downregulates CA1 expression and inhibits aortic calcification.

AZ is an inhibitor of CA1 activity. Many studies have measured the efficiency of AZ in terms of activity inhibition (Mincione et al., 2008; Masini et al., 2013; Solesio et al., 2018). In our study, we found that MTZ and AZ inhibited CA1 expression but did not inhibit other CA members in the AS animal model. In addition, CA1 rather than other CA members had significantly increased expression in human AS tissues. We obtained similar results in our previous studies (Chang et al., 2011; Chang et al., 2012; Zheng et al., 2015). In those studies, AZ inhibited CA1 expression in 4T1 cells (originating from mouse breast tumors) and Saos-2 cells (originating from human osteosarcoma). This means that AZ not only inhibits CA activity but also decreases CA1 expression.

AS plaques form as a result of smooth muscle cell proliferation, and the deposition of cholesterol and other lipids, hydroxyapatite and fibrous connective tissue, and calcium deposition is a critical step in AS (Gamble, 2006). Normally, in adult humans, VSMCs mature and differentiate, with restricted proliferation and migration capabilities. Based on the results of a cellular function examination, upon inhibition of CA1 expression using anti-CA1 siRNA, the proliferation and migration as well as the calcification of rat VSMCs were reduced, while apoptosis was found to be increased. Thus, CA1 may also stimulate smooth muscle cell calcification, proliferation, and migration and suppress apoptosis to accelerate AS pathogenesis.

We successfully established an AS mouse model by feeding ApoE^−/−^ mice a high-fat diet. Sudan IV and Oil Red O staining revealed significantly less plaque formation in the MTZ treatment and preventive treatment groups than in the AS model group. Consistent with these findings, significantly higher serum HDL-c and NO levels were detected in the treatment and preventive treatment groups than in the AS model group. Therefore, MTZ has a therapeutic effect on AS in mice.

Additionally, von Kossa staining showed the presence of substantial atherosclerotic calcification in the aortas of mice from the AS model group, while markedly less calcification was observed in the other three groups. Meanwhile, Western blot analysis demonstrated that the level of CA1 protein was significantly higher in the aortas from the AS model group than in those of the control group, and CA1 expression levels were significantly lower in the MTZ treatment and preventive treatment groups than in the AS model group. CA1 immunohistochemistry also revealed high CA1 expression in the AS model group compared to the control group, and the regions with calcification had high levels of CA1 expression. Compared to the AS model group, CA1 immunoreactivity in the MTZ treatment and preventive treatment groups was significantly reduced. Von Kossa staining and immunohistochemistry also demonstrated colocation of CA1 expression and calcification in human aortic AS tissues. Calcification occurs very early in the process of atherosclerosis. However, it is only able to be detected using imaging modalities when it increases in quantity. Cellular microvesicle release contributes to the development and calcification of atherosclerotic plaque in atherosclerotic plaque formation (Alique et al., 2018; Andrews et al., 2018; Shioi and Ikari, 2018). Our results not only show that CA1 plays a role in AS by regulating calcification but also support the hypothesis that calcification plays important roles in AS progression.

NO has an effect on AS and is a useful index in the AS animal model. Endothelial dysfunction is an important pathogenesis of AS. As an important endothelium-derived relaxation factor, NO plays a role in cardiovascular protection and anti-AS function. Endothelial nitric oxide synthase (eNOS) disorder causes an abnormal production of NO, which may damage endothelial function and trigger AS (Hong et al., 2019). The present study revealed decreased levels of NO in AS animals upon treatment with MTZ, which is in accordance with previous findings.

MTZ could have an effect on organs such as the liver to regulate blood parameters. MTZ is a hepatic insulin sensitizer that lowers blood glucose to treat type 2 diabetes (Konstantopoulos et al., 2012; Simpson et al., 2014). Therefore, the effects of MTZ on blood parameters and plaque histopathology may be independent of the effects of MTZ on plaque calcification. However, another possibility is that inhibition of MTZ in the formation of plaque calcification can subsequently aggravate AS and affect blood parameters.

As shown in the present study, serum IL-6, IFN-γ, GM-CSF, TNF-α, CXCL1/KC, and CCL2/MCP-1 levels were dramatically increased in the AS mouse model. Compared to those in the AS model group, the serum levels of IL-6, IFN-γ, GM-CSF, TNF-α, CXCL1/KC, and CCL2/MCP-1 were significantly reduced in the MTZ treatment and preventive treatment groups, suggesting that MTZ not only suppresses calcification in AS progression but also alleviates the pathogenesis by downregulating the levels of these inflammatory cytokines. Furthermore, VSMCs with AZ treatment or anti-CA1 siRNA transfection also had decreased secretion of IL-6, IFN-γ, GM-CSF, and TNF-α in the culture medium. During AS, multiple inflammatory mediators stimulate VSMC proliferation and migration toward the intima (Bonomini et al., 2016). TNF-α is expressed in the endothelial cells, smooth muscle cells, and macrophages of AS tissues (Ridker et al., 2000). TNF-α promotes the occurrence and development of AS by inducing endothelial cell damage, inhibiting fibrinolysis, promoting coagulation, increasing smooth muscle cell proliferation, and upregulating matrix metalloproteinase expression (Daugherty and Rateri, 2002; Kleinbongard et al., 2010). IFN-γ is an immune-activating factor and mainly affects the inflammatory response and cellular components in plaques in the AS pathogenic process (Koga et al., 2007). GM-CSF is secreted by macrophages, smooth muscle cells, and endothelial cells within the AS plaques. This cytokine is involved in angiogenesis within AS plaques and is closely associated with AS plaque stability and disease progression (Falk et al., 1995; Chen et al., 1999). IL-6 is secreted by vascular endothelial cells, macrophages, and VSMCs and is involved in the formation and stability of AS plaques (Verma et al., 2002; Schuett et al., 2012; Azancot et al., 2015). The chemoattractant effect of MCP-1 leads to the accumulation of numerous macrophages on artery walls and promotes plaque generation and development (Zeng et al., 2003; Wang et al., 2014). MCP-1 accelerates AS progression and is expressed at high levels in macrophages located in AS plaques in ApoE^−/−^ mice (Aiello et al., 1999). CXCL1 plays critical roles in the recruitment of monocytes that leads to AS development (Weber et al., 2008). CXCL1 expressed in the blood vessel promotes macrophage accumulation and induces the capture of monocytes during early AS (Boisvert et al., 2006; Hartmann et al., 2015). Thus, the present results are in accordance with the findings of others and suggest the possibility that the high CA1 expression in AS increases IL-6, IFN-γ, GM-CSF, TNF-α, CXCL1/KC, and CCL2/MCP-1 production to stimulate AS progression. Plaque calcification develops *via* inflammation-dependent mechanisms in AS. Macrophages can undergo two distinct polarization states. Predominantly proinflammatory M1 macrophages promote the initial calcium deposition within the necrotic core of the lesions, which is termed microcalcification. Anti-inflammatory M2 macrophages may facilitate macroscopic calcium deposition, called macrocalcification. Macrocalcification leads to plaque stability, while microcalcification is more likely to be associated with plaque rupture (Shioi and Ikari, 2018). Microcalcifications appear to derive from matrix vesicles enriched in calcium-binding proteins that are released by cells within the plaque (Hutcheson et al., 2014). The present study demonstrated that IL-6, IFN-γ, GM-CSF, and TNF-α had increased production in AS animals and in cultured VSMCs, with induced calcification. MTZ, AZ, and anti-CA1 siRNA decreased the levels of these proinflammatory cytokines. It is possible that CA1 expression plays a role in AS by stimulating calcification and elevating proinflammatory cytokine levels. However, we do not have a sufficient amount of data to demonstrate the involvement of CA1 in microcalcification formation.

In summary, this study found that CA1 was expressed at high levels in calcified human and mouse aortic AS tissues. CA1 expression induced calcification of VSMCs and affected the cell proliferation, apoptosis, migration, and cytokine production. CA1 expression and CA1-mediated calcification were significantly associated with AS progression. MTZ treatment alleviated pathogenic progression in the AS model. By inhibiting CA1 expression with MTZ, AZ, or siRNA, IL-6, IFN-γ, GM-CSF, and TNF-α secretion was significantly decreased in cultured VSMCs and the AS mice. These findings demonstrate the calcification mechanism in AS and suggest that the inhibition of calcification is key in treating AS. MTZ represents a potential treatment for AS.

## Data Availability

All datasets generated for this study are included in the manuscript and the supplementary files.

## Ethics Statement

In this study, all medical research involving human subjects and human materials and data was carried out in accordance with the recommendations of the Declaration of Helsinki. The protocol was approved by the Ethics Committee of Shandong Provincial Qianfoshan Hospital (approval number: 20170607). Prior to sample collection, all patients signed an informed consent form. All animal experiments were conducted in strict accordance with the International Guiding Principles for Biomedical Research Involving Animals set by the World Health Organization and the Regulation of Laboratory Animals in China.

## Author Contributions

XC designed the experiments and wrote the manuscript; LY, HW, YH, and XC performed the experiments; MW, TL, YL, QM, QL, and GZ collected the samples.

## Funding

This study was supported by the Shandong Provincial Key R & D programs (GG201703080038).

## Conflict of Interest Statement

The authors declare that the research was conducted in the absence of any commercial or financial relationships that could be construed as a potential conflict of interest.

## Abbreviations

AS, atherosclerosis; CA1, carbonic anhydrase 1; MTZ, methazolamide; AZ, acetazolamide; VSMC, vascular smooth muscle cell; β-GP, β-glycerophosphate; TC, total cholesterol; TG, triglycerides; LDL-c, low-density lipoprotein cholesterol; HDL-c, high-density lipoprotein cholesterol; NO, nitric oxide; Runx2, runt-related transcription factor 2; BMP2, bone morphogenetic protein 2; ALP, alkaline phosphatase; IFN, interferon; GM-CSF, granulocyte-macrophage colony-stimulating factor; TNF-α, tumor necrosis factor-α; CXCL1/KC, (C-X-C motif) ligand 1/keratinocyte-derived chemokine; CCL2/MCP-1, C-C motif chemokine ligand 2 (CCL2)/monocyte chemoattractant protein 1 (MCP-1).

## Supplementary Materials

Figure S1 The effect of MTZ on body weight of ApoE^−/−^ mice. *N* = 10 for each group. Data are shown as the means ± SEM.

Figure S2 The mRNA expression level of each CA member in AS animal model aortic tissue. Data are shown as the means ± SEM. **P*< 0.05, ****P*< 0.001.
