# Supplementary figures and images for: Carbonic Anhydrase 1-Mediated Calcification Is Associated With Atherosclerosis, and Methazolamide Alleviates Its Pathogenesis
Source: Front Pharmacol. 2019 Jul 10;10:766. doi: 10.3389/fphar.2019.00766 (PMC6635697; doi:10.3389/fphar.2019.00766)

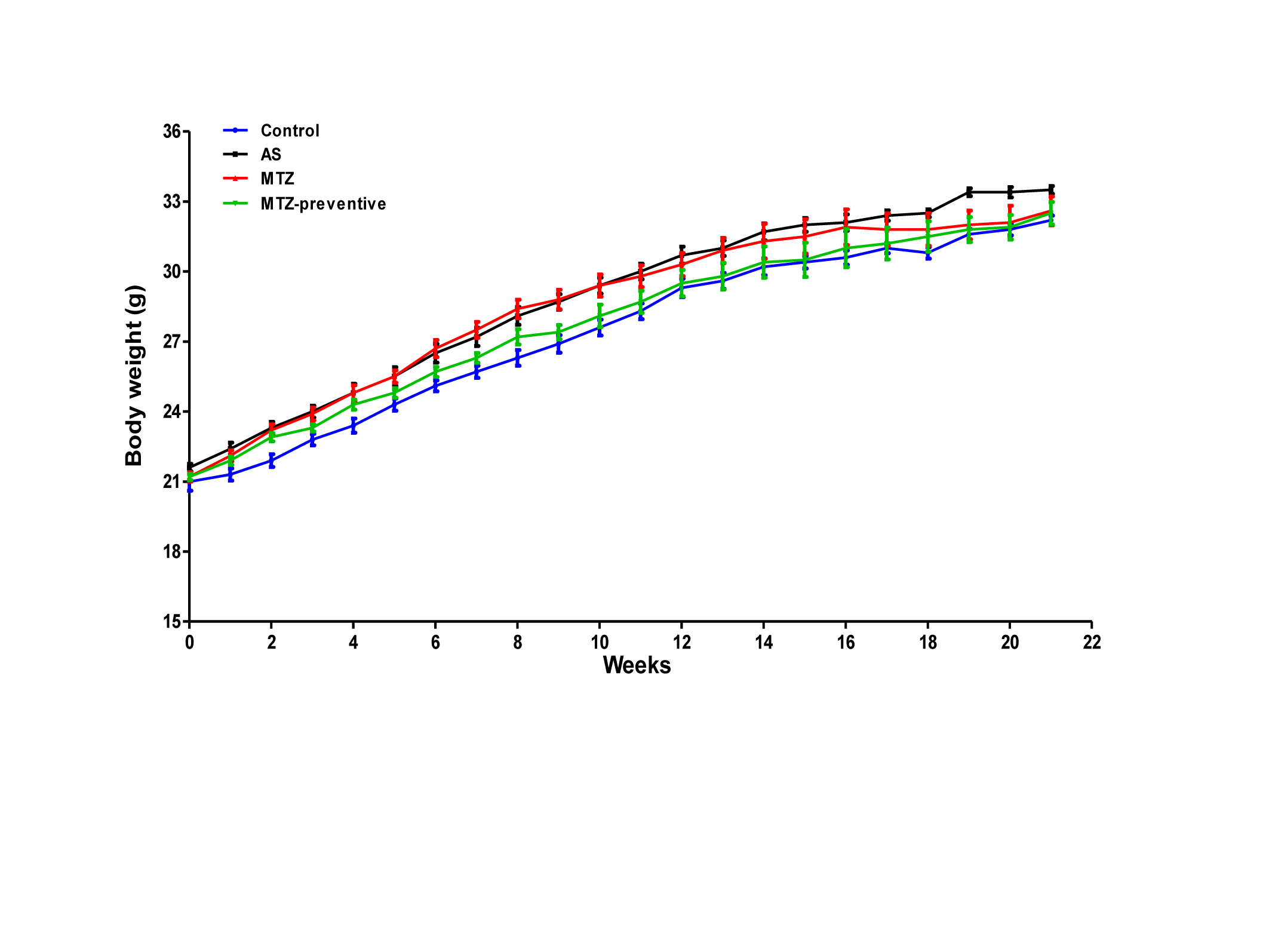

Supplement: Figure S1 — The effect of MTZ on body weight of ApoE−/− mice. N = 10 for each group. Data are shown as the means ± SEM. [file Image_1.tif]

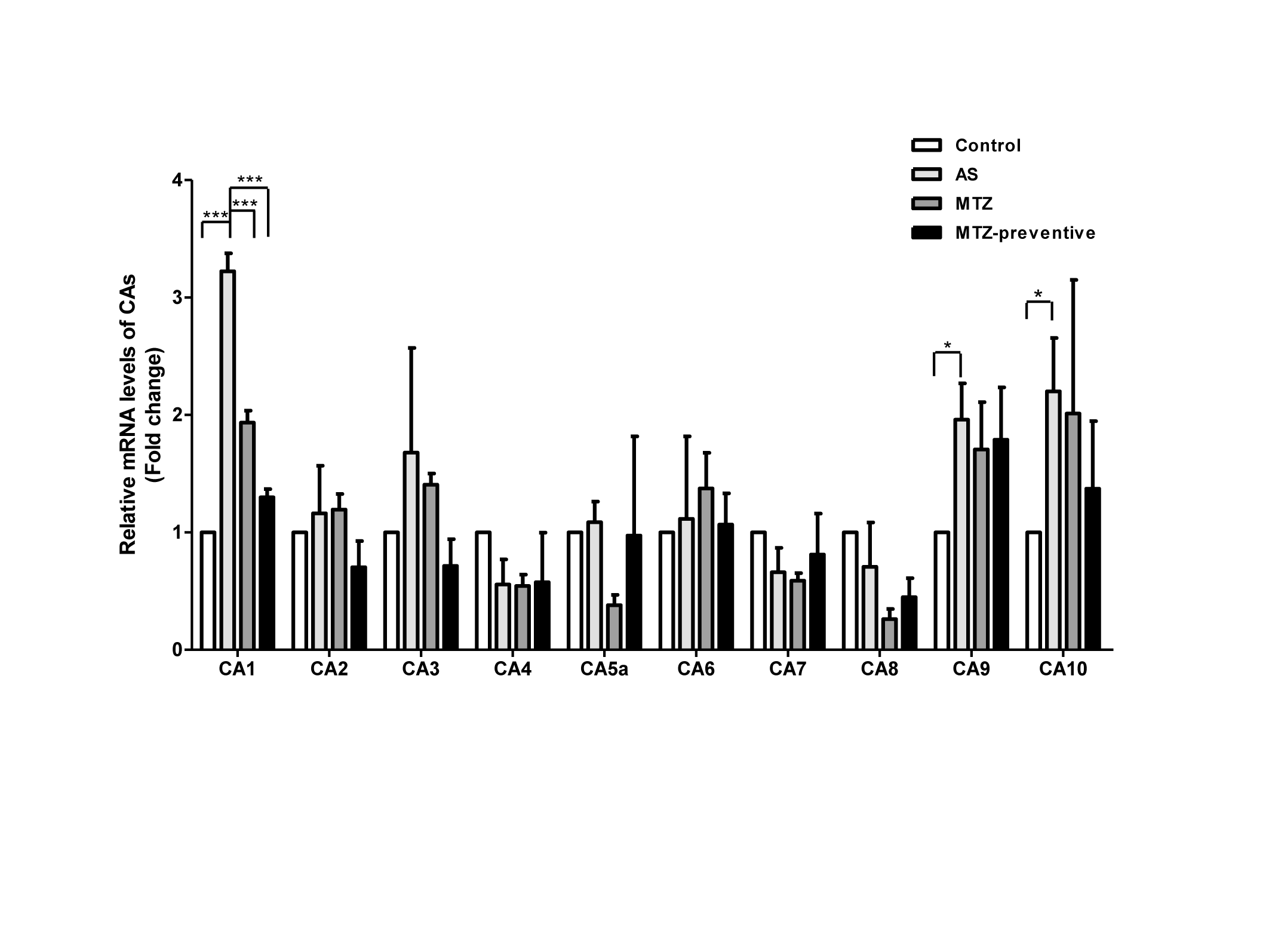

Supplement: Figure S2 — The mRNA expression level of each CA member in AS animal model aortic tissue. Data are shown as the means ± SEM. *P< 0.05, ***P< 0.001. [file Image_2.tif]
