# Supplementary material for: Carbonic Anhydrase 1-Mediated Calcification Is Associated With Atherosclerosis, and Methazolamide Alleviates Its Pathogenesis
Source: Front Pharmacol. 2019 Jul 10;10:766. doi: 10.3389/fphar.2019.00766 (PMC6635697; doi:10.3389/fphar.2019.00766)
Supplement: Supplementary file 3 [file Table_1.docx]

**Supplementary Table 1** | **Patient** **clinical data**

**Patient Diagnosis Sex Age (years) Collection date AS and calcification**

1 ascending aortic aneurysm male 56 2017.01.16 yes

2 ascending aortic aneurysm male 37 2017.09.04 yes

3 ascending aortic aneurysm male 46 2017.10.20 yes

4 aortic dissection（Debakey I） male 51 2017.12.16 yes

5 aortic dissection（Debakey I） female 60 2017.12.19 yes

6 aortic dissection（Debakey I） female 54 2018.01.29 yes

7 aortic dissection（Debakey I） female 52 2018.07.01 yes
