# Supplementary material for: Carbonic Anhydrase 1-Mediated Calcification Is Associated With Atherosclerosis, and Methazolamide Alleviates Its Pathogenesis
Source: Front Pharmacol. 2019 Jul 10;10:766. doi: 10.3389/fphar.2019.00766 (PMC6635697; doi:10.3389/fphar.2019.00766)
Supplement: Supplementary file 4 [file Table_2.docx]

**Supplementary Table 2 | Primer sequences for real-time PCR**

| **Gene** | **Forword (5’-3’)** | **Reverse (5’-3’)** |
| --- | --- | --- |
| ALP (rat) | TCATAATTCCAGGCCGAACCA | GGCCATCTTAGCAGCAACTTTCA |
| BMP2 (rat) | CAGCGGAAGCGTCTTAAGTCCAG | GGCATGGTTGGTGGAGTTCAGG |
| CA1 (human) | CTGACAGCTACAGGCTCTTTC | CTACGTGAAGCTCGGCAGAAT |
| CA2 (human) | ATGGTCATGCTTTCAACGTGG | TGTCCATCAAGTGAACCCCAG |
| CA3 (human) | AAGACCTGCCGAGTTGTATTTG | CCCCTCTCAGCATTGACCTATC |
| CA4 (human) | CTGGTGCTACGAGGTTCAAGC | GAAGAAGAAGCGTCCCAGTTT |
| CA5a (human) | GTGCATGGCAAACCAGCAATA | CCGCTTCATAGGAGACCCTG |
| CA6 (human) | GTACTGGCAGCCTTCGTTGAG | TGGTAGGTGTAGTAGTGCTGG |
| CA7 (human) | TGACAGCGATGACCGAACC | ACTTCTTGGCATTCCAGTGAAC |
| CA8 (human) | GGAAGTCCAAAACAATACCTTGC | TGGTGAGAGAGCCTTCATACAC |
| CA9 (human) | TTTGCCAGAGTTGACGAGGC | GCTCATAGGCACTGTTTTCTTCC |
| CA10 (human) | TCATCGTCTGCATATCAGCTCA | GTTCACCAATCCCCAGAAAGAAG |
| CA1 (mouse) | TCTGAAGCCATCTCCAAGGC | ATGGGGCTCGTTTTCCCTTAG |
| CA2 (mouse) | ACTGGACATACCCTGGCTCT | TCGCTGCTGACAGTAATGGG |
| CA3 (mouse) | TGCCGGGACTATTGGACCTA | GCTCACAGTCATGGGCTCTT |
| CA4 (mouse) | GGCCCCCTCTACTGAAGACT | GGTCATAGCCGACGAGGATG |
| CA5a (mouse) | GCAGAGGAACACAGCAACTG | TAGGAGACCCTGAGTGGTGC |
| CA6 (mouse) | GGATGATGGCGTGGGAGAAA | TCCTCCGTCTTCACGTCGAT |
| CA7 (mouse) | GGAGATGAGCACCCAAGCAT | TCAGGGAGCCAGGATAGGTC |
| CA8 (mouse) | TCTGCGGGATTACTGGGTCT | ACATGTGTCCTCAGCCTTGG |
| CA9 (mouse) | GAAGAGGATGCACCTGAAGAGG | GCCTCCATAGCTCCAATGACT |
| CA10 (mouse) | AATCCATGAAGGCTGGTGGG | GAGGTGTCAGAAAGGGGTCG |
| GAPDH (human) | CAGAACATCATCCCTGCCTCTAC | TTGAAGTCAGAGGAGACCACCTG |
| GAPDH (mouse) | GGACACTGAGCAAGAGAGGC | TTATGGGGGTCTGGGATGGA |
| GAPDH (rat) | TCTCTGCTCCTCCCTGTTCT | ATCCGTTCACACCGACCTTC |
| Runx2 (rat) | CATGGCCGGGAATGATGAG | TGTGAAGACCGTTATGGTCAAAGTG |
