# Supplementary material for: Carbonic Anhydrase 1-Mediated Calcification Is Associated With Atherosclerosis, and Methazolamide Alleviates Its Pathogenesis
Source: Front Pharmacol. 2019 Jul 10;10:766. doi: 10.3389/fphar.2019.00766 (PMC6635697; doi:10.3389/fphar.2019.00766)
Supplement: Supplementary file 5 [file Table_3.docx]

**Supplementary Table 3** | **Clinical information associated with tissue arrays**

**NO. Type Sex Age (years) Pathological diagnosis**

1 normal female 21 coronary artery tissue

2 normal female 40 coronary artery tissue

3 normal female 50 aorta tissue

4 normal male 28 aorta tissue

5 normal male 40 large artery tissue

6 normal male 2 large artery tissue

7 normal female 43 large artery tissue

8 normal female 27 artery tissue

9 disease male 33 early atherosclerosis of internal carotid artery tissue

10 disease male 28 early atherosclerosis of large artery tissue

11 disease female 50 early atherosclerosis of abdominal aorta tissue

12 disease male 48 atherosclerosis of abdominal aorta tissue

13 disease male 47 atherosclerosis of coronary artery tissue

14 disease male 50 atherosclerosis of carotid artery tissue

15 disease male 38 atherosclerosis of large artery tissue

16 disease male 42 atherosclerosis of artery tissue
